# Out-of-hours discharge from intensive care, in-hospital mortality and intensive care readmission rates: a systematic review protocol

**DOI:** 10.1186/s13643-015-0081-8

**Published:** 2015-07-16

**Authors:** Sarah A. Vollam, Susan J. Dutton, Duncan Young, Peter J. Watkinson

**Affiliations:** Nuffield Department of Clinical Neurosciences, University of Oxford, Oxford, UK; Oxford Clinical Trials Research Unit, Centre for Statistics in Medicine, Nuffield Department of Orthopaedics, Rheumatology and Musculoskeletal Sciences, University of Oxford, Oxford, UK; Kadoorie Centre for Critical Care and Trauma Research and Education, John Radcliffe Hospital, Headley Way, Oxford, OX3 9DU UK

**Keywords:** Intensive care, Critical care, High dependency unit, Mortality, Out-of-hours, Systematic review, Meta-analysis

## Abstract

**Background:**

Most patients are discharged from an intensive care unit with an expectation that they will survive their hospital stay, yet these patients have high subsequent in-hospital mortality. Patients are frequently discharged from an intensive care unit to a lower level of hospital care in the evenings and at night (out-of-hours). By affecting the care that patients receive, out-of-hours discharge may alter post-intensive care in-hospital mortality rates.

**Methods/design:**

Two searches will be conducted—the first a general search for all factors associated with post-intensive care in-hospital mortality and a second focused specifically on out-of-hours discharges. Searches will be performed in multiple databases, including Medline, Embase, Web of Knowledge, Cumulative Index of Nursing and Allied Health Literature (CINAHL) and the Cochrane Library. OpenGrey will also be searched, to ensure any unpublished ‘grey’ data are accessed. Language and date restrictions will not be applied. Assessment for inclusion and data extraction will be undertaken by two independent reviewers. Methodological quality will be assessed using the ACROBAT-NRSI tool. The primary outcome measure will be post-intensive care in-hospital mortality. To provide a clearer picture of this problem, studies reporting readmission to the intensive care unit (ICU) will also be included, even in the absence of report of in-hospital mortality.

The primary outcome data will be synthesised and summarised using a random-effects meta-analysis. Where possible, subgroup meta-analyses will assess associated factors such as discharge destination, palliative care discharges and severity of illness scores.

**Discussion:**

To the best of our knowledge, a systematic review of the association of out-of-hours discharge with in-hospital mortality has never been undertaken. Synthesis of the available information is important because out-of-hours discharge remains common and, if associated with post-intensive care unit mortality, is highly amenable to system change.

**Systematic review registration:**

PROSPERO CRD42014010321

**Electronic supplementary material:**

The online version of this article (doi:10.1186/s13643-015-0081-8) contains supplementary material, which is available to authorized users.

## Background

### Rationale

Discharge from an intensive care unit (ICU), rather than representing recovery from the life-threatening part of an illness, is for many patients only the start of a high-risk journey. Subsequent in-hospital mortality rates are reported to be 5.9–13.3 % in multi-centre studies [1, 2], representing around a third of all ICU-associated mortality. These findings compare unfavourably with in-hospital mortality in other groups considered high-risk such as patients after upper gastrointestinal surgery (2.4 %) or cardiothoracic surgical patients (2.7 %) [3, 4]. In fact, in-hospital mortality following discharge from intensive care is at least comparable with mortality for the entire hospital stay (including deaths on intensive care) for patients admitted with acute exacerbations of chronic obstructive pulmonary disease (COPD) (7.5 %) [5]. As early as the 1980s, the need to investigate the discharge and subsequent management of patients who survive ICU was acknowledged [6–8]. As out-of-hours discharge from an ICU could be considered a marker of premature discharge (the patient is discharged before they are ready because of bed pressures for example) [9] or because discharge out-of-hours may result in a relatively high-intensity patient arriving in area with less staff than in the daytime, resulting in decreased care [10], some authors have looked specifically at the effect of out-of-hours discharge from an ICU as a factor in this high post-ICU mortality rate [1, 2, 11]. There are also many studies which have retrospectively interrogated intensive care databases which may contain information on the effect of out-of-hours discharge [12, 13]. To the best of our knowledge, a systematic review of the association of out-of-hours discharge with in-hospital mortality, incorporating data from both of these two types of studies, has never been undertaken. Synthesis of the available information is important because out-of-hours discharge remains common [14] and, if associated with post-ICU mortality, is highly amenable to system change.

### Objective

This review aims to determine the effect of out-of-hours discharge in comparison to in-hours discharge on post-ICU in-hospital mortality in survivors of treatment on an ICU. Where possible, factors associated with this effect, such as discharge destination, definition of out-of-hours and inclusion of palliative care discharges will also be examined.

### Strengths and limitations

This review will be the first to synthesise the evidence on the effect of out-of-hours discharge from ICUs on hospital mortality.

It may be limited by differences in the definitions of ‘out-of-hours’ and ‘in-hours’ between studies (and between institutions in which the research has been undertaken). It may also be limited by different definitions of discharge destination (high dependency unit and ward level). As with all such analyses, it may be limited by the quality of the available data.

## Methods/design

This protocol has been developed using the PRISMA (Preferred Reporting Items for Systematic Reviews and Meta-Analyses) [15], PRISMA-P (Preferred Reporting Items for Systematic Reviews and Meta-Analyses—Protocol-specific) [16] and MOOSE (Meta-analysis Of Observational Studies in Epidemiology) guidelines [17] where applicable.

### Eligibility criteria

To be included, patients must have been discharged alive from a general surgical, medical or mixed intensive care unit to a lower level of in-hospital care (high dependency or ward level) and at discharge must have been defined as discharged out-of-hours or in-hours. All patient ages (≥16 years) and conditions will be included. The primary outcome measure will be post-ICU in-hospital mortality, and studies reporting this outcome will be included. To provide a clearer picture of this problem, studies reporting readmission to ICU will also be included, even in the absence of report of in-hospital mortality. We will include original studies which use quantitative methods of data collection and analysis. Where appropriate, we will use review articles including systematic reviews to facilitate identification of original data. Date and language restrictions will not be applied, and every effort will be made to access translations of potentially relevant articles not in English. Where possible, we will include both published and unpublished data.

### Data sources

Searches will be performed in multiple databases, including Medline, Embase, Web of Knowledge, Cumulative Index of Nursing and Allied Health Literature (CINAHL) and the Cochrane Library. OpenGrey will also be searched, to ensure any unpublished ‘grey’ data are accessed.

### Search strategy

The design of this search strategy will be guided by a medical librarian, who will assist in the conduct of these searches. Two searches will be conducted—the first a general search for all factors associated with post-ICU in-hospital mortality and readmission to ICU and a second focused specifically on out-of-hours discharges. The two searches will be conducted as some studies may report out-of-hours discharge as one of many variables contributing to post-ICU in-hospital mortality, and therefore, a more focused search would miss these, particularly if the findings are non-significant and therefore unlikely to feature in the abstract. Also, where studies are found in the general search which report multiple variables associated with post-ICU in-hospital mortality but which do not report the effect of out-of-hours discharge, authors will be contacted to discover whether this information was extracted, but not published. We anticipate that studies reporting readmission to ICU will also report in-hospital mortality, but both search terms will be included to ensure we capture all relevant studies. An initial detailed search strategy for Medline is included as an additional file (Additional file 1) and will be adapted where necessary to the database being searched. Search terms will include (mortality OR death* OR die OR died OR readmission), (ITU OR ICU OR AICU OR intensive care OR critical care OR intensive therapy unit), (post OR after OR following OR discharge OR ward* OR inhospital OR ‘in hospital’ OR ‘transfer* from’) and (‘out-of-hours’ OR off-hour OR night*time OR evening). Where possible, terms will be ‘exploded’ and MeSH terms will be used. Once both searches have been conducted, the findings will be pooled and duplicates removed. The focused search will act as a second check to ensure no pertinent studies are missed.

Once the initial searches have been performed and a list of studies for inclusion has been agreed, we will conduct further searches using relevant keywords (using Medline) from papers included from the initial search and citation searches (using Web of Knowledge) for each paper.

### Study selection

Results will be reviewed in three stages—at title, at abstract and at full text.

Stage 1: Search results will be screened by title by two independent researchers and either rejected as obviously not relevant or selected for abstract review. Where disagreement occurs at this stage, the article will remain for consideration at the abstract stage.

Stage 2: Articles selected at title will have abstracts reviewed by two independent researchers and either rejected as obviously not relevant or selected for full text review. Any discrepancies between the two researchers will be discussed and agreed with a third reviewer. Where any doubt remains, the full text will be retrieved.

Stage 3: Full text articles for review will be collated and will be assessed independently by two reviewers. Studies which otherwise meet inclusion criteria will be excluded if

- They included patients who were predominantly discharged from specialist intensive care units (for example cardiothoracic or neurosurgical units) or were restricted to a specialist patient group (for example liver transplant patients).
- Post-ICU mortality cannot be identified from whole hospital stay mortality.
- Follow-up was discontinued before hospital discharge.

**Fig. 1:**
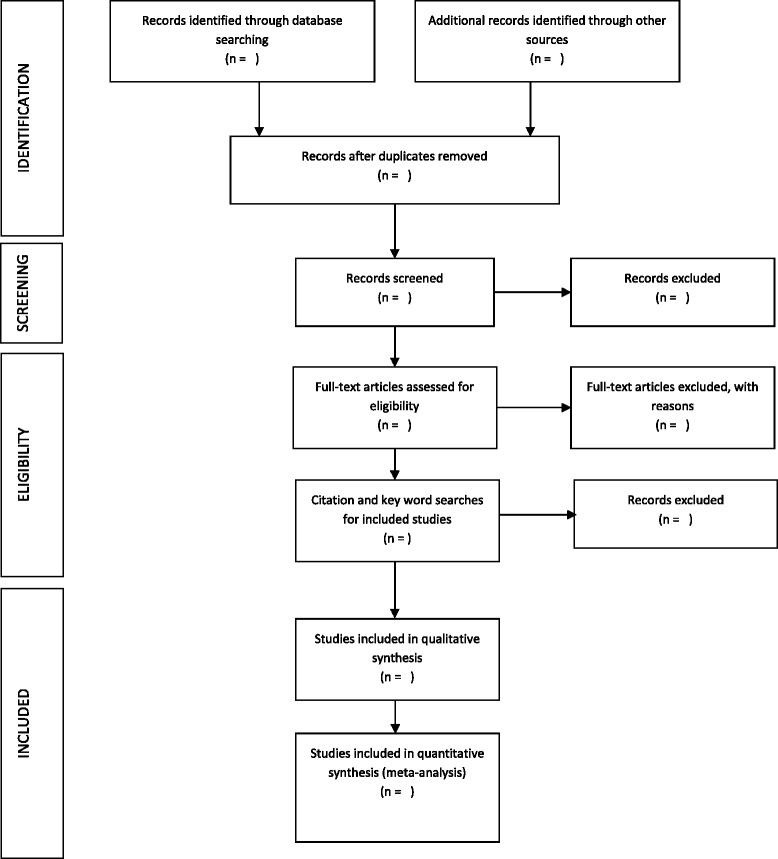
PRISMA flowchart

As before, any discrepancies between reviewers will be discussed with a third reviewer. Where eligibility cannot be ascertained, the authors of the study will be contacted for clarification. An overview of the selection process is shown in Fig. 1.

### Data collection process

After conducting the searches, the results will be exported to an independent database and merged and duplicates automatically identified and removed, as described above. Each team member will receive a copy of this final database, using a reference manager software (Endnote, Thomson Reuters, www.endnote.com).

Data for each study will be extracted by two researchers using data extraction tables which will be piloted prior to use. These data will include the type of publication, date of publication, study type, setting, numbers of patients, eligibility criteria, missing data, definitions of in-hours and out-of-hours and main findings: numbers of deaths in each group, effect sizes (relative risk or odds ratio and their CIs), population and cohort data, main conclusions and data to allow risk of bias analysis (see Table 1). Where there is lack of clarity in the data extracted, this will be sought from the authors. Where studies do not report participant-level data, this will be sought from the authors.

**Table 1:** Data extraction categories

Patients/population	Age, sex, surgical status (elective, emergency, none), severity of illness assessment. Availability of high-dependency care within ICU or in discrete unit, ICU type
	Assessment of occupancy
	Assessment of premature discharge
Intervention	Proportion discharged ‘in-hours’. Definition of in-hours
	Discharge destination (level of subsequent care)
	Proportion of discharges deemed ‘premature’
Comparison	Proportion discharged ‘out-of-hours’. Definition of out-of-hours
	Discharge destination (level of subsequent care)
Outcome assessment	Mortality associated with out-of-hours discharge
	Data source for mortality
	Coding of palliative care patients
	Missing data
	Readmission rate
	Data source for readmission rate associated with out-of-hours discharge
	Severity score assessment of in-hours versus out-of-hours groups
Study	Study design, number of sites, authors, publication year, country, duration
	Primary endpoint (where stated) or main focus (time of discharge versus factors associated with outcome post-discharge, other)
Quality assessment	ACROBAT-NRSI criteria
	Sources of participants
	Follow-up time
	Completeness of data
	Adjustment for potential confounders
	Further subjective assessment in relation to heterogeneity of studies
	Method of severity of illness assessment
	Method of risk adjustment
	Risk-adjusted results

### Protocol amendments

To ensure transparency of process, any amendments to this protocol will be documented separately with date, description and rationale. Amendments will not be made to the main body of the protocol, as suggested by the PRISMA-P guidelines [16].

### Analysis

#### Risk of within-study bias assessment

Once all searches have been completed, the included studies will be assessed for quality using the Cochrane Risk Of Bias Assessment Tool: for Non-Randomized Studies of Interventions (ACROBAT-NSRI) [18]. This scale examines bias in seven domains through four stages of a study: pre-intervention, at-intervention and post-intervention. The final output offers five levels of risk of bias: low risk, moderate risk, serious risk, critical risk and no information on which to base a judgement.

It is not anticipated that results will be used to weight studies in the meta-analysis, but the results will be used to aid assessment of the overall results. This will be addressed in the discussion. Bias assessment of individual papers will be made available in the final publication.

#### Synthesis of results

From extracted data, the mortality rate of out-of-hours discharges and that of in-hours discharges will be compared over all included studies. As there are likely to be different definitions of out-of-hours discharge between studies and some studies may include different types of discharge criteria, it is expected that effects may vary between studies. Therefore, data will be synthesised and summarised using a random-effects meta-analysis with the mortality risks expressed as relative risk, showing the mean effect and 95 % confidence intervals, with the significance level (*p* value). The DerSimonian and Laird Method of computing the between-studies variance will be utilised. Results will be displayed in a forest plot using either RevMan (http://tech.cochrane.org/revman) or using the Stata metan procedure (StataCorp LP). This process will also be followed to analyse readmission to ICU for out-of-hours and in-hours discharges.

#### Assessment of heterogeneity for meta-analysis

Based on our current knowledge of the available data, it is anticipated that meta-analysis will be possible for some if not most studies. Data will be aggregated at the level of individual studies. An assessment of heterogeneity will be made (using both the *χ*^2^ test and the *I*^2^ statistic). Sensitivity analysis will be carried out by repeating the random-effects meta-analysis omitting studies of different quality or risk of bias.

#### Risk of bias across studies

Visual assessment of funnel plots and Egger’s regression will be used to assess publication bias. The GRADE (Grading of Recommendations Assessment, Development and Evaluation) methodology will be used to report the overall strength of the review as high, moderate, low or very low [19].

#### Subgroup analysis

If sufficient numbers of studies differentiate between discharge destinations (ward or ‘high dependency’ area), a random-effects subgroup meta-analysis will be undertaken. Other potential subgroup analyses that will be undertaken if there are sufficient studies will include different definitions of out-of-hours and in-hours, inclusion or exclusion of patients discharged for palliative care (or other similar limitation of treatment) and whether intensive and high-dependency care were provided within the same physical facility. In addition, reflecting the potential for change in practice across the time spread of studies, differential effects over time will be considered and analysed if sufficient data are available. These analyses will be presented as before but for the individual subgroups and combined overall.

## Discussion

This systematic review will synthesise current available evidence on whether out-of-hours discharge affects post-ICU in-hospital mortality, a synthesis which has not previously been undertaken. In undertaking the proposed subgroup meta-analyses, associated considerations (such as inclusion of palliative care discharges and level of care at discharge destination) will also be examined. Whilst, as with all systematic reviews, the findings may be limited by the quality, comparability and potential biases within the available literature, undertaking the analysis remains important. Preventing out-of-hours discharge impacts out-of-hours admissions, where delay may also have deleterious consequences or require costly spare capacity within intensive care units. It is therefore only rational to prevent out-of-hours discharge if there are significant deleterious consequences to these patients. Conversely, if out-of-hours discharge does present a significant patient risk, it is highly amenable to system change.

### Study registration

This systematic review has been registered with PROSPERO—the international prospective register of systematic reviews, registration number: CRD42014010321.

## Additional file

Additional file 1:
**Sample search strategy.** An initial detailed search strategy for Medline.

## Abbreviations

ACROBAT-NRSI: A Cochrane Risk Of Bias Assessment Tool: for Non-Randomized Studies of Interventions
AICU: adult intensive care unit
CI: confidence interval
CINAHL: Cumulative Index of Nursing and Allied Health Literature
COPD: chronic obstructive pulmonary disease
GRADE: Grading of Recommendations Assessment, Development and Evaluation
ICU: intensive care unit
ITU: intensive therapy unit
MeSH: medical subject headings
MOOSE: Meta-analysis Of Observational Studies in Epidemiology
PRISMA: Preferred Reporting Items for Systematic Reviews and Meta-Analyses
PRISMA-P: Preferred Reporting Items for Systematic Reviews and Meta-Analyses—Protocol-specific
