# Supplementary material for: Out-of-hours discharge from intensive care, in-hospital mortality and intensive care readmission rates: a systematic review protocol
Source: Syst Rev. 2015 Jul 16;4:93. doi: 10.1186/s13643-015-0081-8 (PMC4502566; doi:10.1186/s13643-015-0081-8)
Supplement: Additional file 1: — Sample search strategy. An initial detailed search strategy for Medline. [file 13643_2015_81_MOESM1_ESM.pdf]

Supplementary File 1.

Sample Search Strategy

The search strategy will be adapted according to the database being searched, but will generally include the following terms:

1. MORTALITY
2. *DEATH
3. READMISSION
4. (mortality OR death* OR die OR died)
5. 1 OR 2 OR 3 OR 4
6. *INTENSIVE CARE
7. *INTENSIVE CARE UNITS
8. *CRITICAL CARE
9. "intensive care"
10. "intensive treatment"
11. "intensive therapy"
12. "critical care"
13. "critical* ill*"
14. (ITU OR ICU OR AICU)
15. 6 OR 7 OR 8 OR 9 OR 10 OR 11 OR 12 OR 13 OR 14
16. *PATIENT DISCHARGE
17. discharge*
18. (post OR after OR following)
19. (ward* OR inhospital OR "in hospital")
20. "transfer* from"
21. 16 OR 17 OR 18 OR 19 OR 20
22. “out of hours”
24. off-hour
25. night-time
26. 22 OR 23 OR 24
27. 5 AND 15 AND 21 AND 25

For initial searches, no limits will be set.
